# Body Expression-Based Intervention Programs for Persons with Intellectual Disabilities: A Systematic Review

**DOI:** 10.3390/ijerph17207569

**Published:** 2020-10-18

**Authors:** María-Jesús Lirola, Gerardo Ruiz-Rico, Antonia-Irene Hernández-Rodríguez, María-Esther Prados-Megías

**Affiliations:** Department of Education, Educational Sciences Faculty, University of Almería, 04120 La Cañada de San Urbano, Spain; mariajesus.lirola@ual.es (M.-J.L.); ruizrico@ual.es (G.R.-R.); eprados@ual.es (M.-E.P.-M.)

**Keywords:** inclusion, non-verbal language, physical activity, Down syndrome, autism spectrum disorder

## Abstract

The benefits of physical exercise on human health are widely known. However, the level of physical activity in the majority of the population is considered insufficient. People with intellectual disabilities (ID), in particular, show this lack of physical activity to a greater extent. It has been noted that the promotion of activities focused on corporal expression (CE) stimulates the motivation to carry out physical exercise in this population group. The aim of this study was to carry out a systematic review of the last ten years of CE programs carried out in people with ID. The criteria established in the PRISMA statement were followed in the literature search. The databases consulted were Scopus, Web of Science, Pubmed, PsycInfo and Elton B. Stephens Company (EBSCO). Eight exclusion criteria were established for the selection of articles. For the analysis of the selected research, three quality criteria for interventions were established. Subsequently, a summary table with the characteristics of each study was made. In conclusion, it can be stated that CE programs in people with ID report benefits at both physical and psychosocial levels. It is necessary to extend this approach for the promotion of healthy physical activity that advocates social inclusion.

## 1. Introduction

In modern society, sedentary life is one of the most common risks affecting a large part of the population, especially the most vulnerable groups, and in particular people who have been diagnosed with some form of intellectual disability (ID) [1]. ID is defined as the presence of certain limitations regarding the functioning of the brain and the manifestation of behaviors poorly adaptive to the environment, with subjects having to present these difficulties before the age of 18 to be diagnosed [2]. There are different studies [1,3,4,5] that show how lack of movement and physical activity affect mental and physical health and especially in people with ID. Studies generally reveal that sedentary habits and behaviour are positively and significantly associated with high rates of chronic disease in this population, such as diabetes, hypertension, cardiovascular disease, obesity, osteoporosis and even multi-morbidity [6,7,8].

In this sense, as pointed out by Oviedo et al. and Jobling [9,10], one of the most important reasons why people with ID develop sedentary behaviour is related to lack of motivation to practice physical activity, hence the importance of implementing specific programs for this group to encourage them to engage in physical activity as a habit in their daily lives [11]. However, current research reveals a need to apply such programs during the transition from compulsory schooling to adulthood, since it is at this stage that there is a greater risk of sedentarism in this population [12]. 

Nevertheless, it is relevant to consider what the needs and motivations of people with ID are in order to promote their adherence to physical activity practice as these may differ from other population groups [13]. It is important, therefore, to consider their preferences when designing and implementing physical activity promotion campaigns. An interesting strategy would be to carry out activities that work especially on social relations, and on communication in any of its facets, since this promotes motivation towards participation [14,15]. Some studies reflect that the design of these activities can be complex, particularly because people with ID sometimes exhibit inappropriate social-communicative behaviors, possibly and in some cases due to their difficulties in recognizing emotional and facial expressions and their misperceptions of social situations [16,17,18]. 

In contrast, more recent research shows how people with ID have good paralinguistic abilities in adjusting or moderating their behavior to socio-emotional requirements. These skills are understood as components of non-verbal communication, which includes phonetic units, facial expressions, corporal gestures, hand gestures, etc., and are used directly to facilitate communication [19], thereby achieving better adaptability to the social skills required by context than that shown by people without ID [20,21]. 

One of the most important contributions that combines the acquisition of physical, communicative-relational, expressive and movement skills has been made by Corporal Expression (CE). CE was introduced as a field of study in the middle of the 20th century, incorporating practices from different orientations from the Social, Psychological-Therapeutic, Scenic-Artistic, Philosophical-Metaphysical and Pedagogical-Educational fields [22] and influences from other corporal disciplines such as theatre, dance or music, either from the Eastern or Western world [23,24]. CE as a discipline is present in different educational stages via Physical Education, but it is also an important tool in intervention programs for social and community integration and in psychotherapy [25]. This discipline, in its multiple manifestations and areas of application [26], requires the development of physical, artistic and expressive capacities through different types of language based on the emotional heritage of the users. The different expressive resources (e.g., non-verbal language, gestures, mime, dance, body awareness, expressive and musical games, dramatization, improvisation and interpretation) enhance experience and psychological training by improving relationships and communication with the environment [27,28,29,30,31].

Studies state that CE is one of the tools that most promotes and helps the inclusion and integration of groups with ID in different environments and contexts. The study by Shih and Chiu [32] indicates that some of the contents of CE, such as dance or choreography, increase motivation and adherence to sports practice in people with ID. Another important characteristic related to adherence to physical activity practice is improvement in the perception of social relationships. The practice of CE activities by people with ID reduces communication and social barriers, facilitating participation and inclusion processes [33,34]. 

In this sense, research in the field of social and educational pedagogy highlights the need to reduce stigma and blur categorizations in people with ID [35]. Along these lines, the works of these authors on the body and its expressive capacities open up paths for new knowledge from the perspective of a sensitive pedagogy, i.e., by considering that “the other person is radically different from me (and that precisely because of this they cannot be myself) is going to be materialized in what some have called “abnormal”, different, rare, queer, crip, freak, etc.” [35]. This type of positioning has to become the central component of CE proposals which should consider otherness as one of the central axes. 

Systematic reviews have recently been conducted on the influence of sports activities on the physical condition of people with ID, and the results confirm the improvement in health status and self-perception [36,37]. A literature study on the construct of CE and its different nominations and applications [26] also shows the growing number of publications in this area of knowledge. Notwithstanding, to date there has been no literature review conducted that reveals the benefits of intervention programs based on the field of corporal expression in the target population of this study. 

This article addresses two objectives; on the one hand, to review and evaluate the existing scientific literature related to the contributions that different CE programs have made to people with ID, and on the other hand, to analyze the results of such research and open new topics within the field of CE with people with ID.

## 2. Materials and Methods 

Following the principles established by the PRISMA declaration [38] for the elaboration of systematic reviews, a literature review was carried out in the following electronic databases: Scopus, Web of Science (WoS), PubMed, PSycINFO, and Elton B. Stephens Company (EBSCO) (including ERIC, CINAHL complete, Medline, Education Source, and PsicoDoc), as a strategy to compile different research studies conducted in the period between January 2010 and April 2020, to analyze the intervention programs implemented in the population with intellectual disability whose main component is corporal expression work, using the following descriptors or key words in the English language: intellectual disability, intellectual functional diversity, intellectual development disorder, body expression, corporal expression, dramatization, non-verbal communication, dance. The search was limited to texts written in English or Spanish. One of the first three descriptors was requested using OR between them, as well as the last five keywords; furthermore, both parts were combined by conjugating the Boolean descriptor AND. The review of the information was completed using the bibliographic references found in the previous searches. As inclusion criteria, articles on intervention were considered, without discriminating by minimum sessions that described performance made by people with intellectual disability without taking into account a determined age range, or the severity of such disability. All interventions had to be corporal expression programs. These articles were reviewed and evaluated thoroughly. All studies that contained some of the key words in their title but whose content did not address the relationship set out in the objective of this study were dismissed.

### 2.1. Assessment of the Quality of the Search

The methodological validity of the papers was assessed by two independent reviewers before their inclusion in the review. Each eligible study was evaluated by two new reviewers independently, all of them competent in English and Spanish. A consensus meeting was held to resolve any disagreements on applicability and quality between the reviewers and a third independent reviewer was consulted when the two reviewers could not reach agreement. The reviewers assessed the risk of bias, including the evaluation of each study for the selection processes, sample size, methods, measurement and study results, as well as effect estimates and confidence intervals (CI), when possible.

### 2.2. Data Extraction

A total of seven exclusion criteria were established for the selection of articles to be studied:

- C1: Excluded for not being in English or Spanish
- C2: Excluded for being out of the period 2010–present
- C3: Excluded because they are abstract only and/or do not provide sufficient data
- C4: Excluded because in the study no intervention was performed
- C5: Excluded because they are not performed with persons with intellectual disabilities
- C6: Excluded because they are not related to the practice of corporal expression in a concrete way
- C7: Excluded because they are related to persons with intellectual disabilities in medical fields

**Figure 1:**
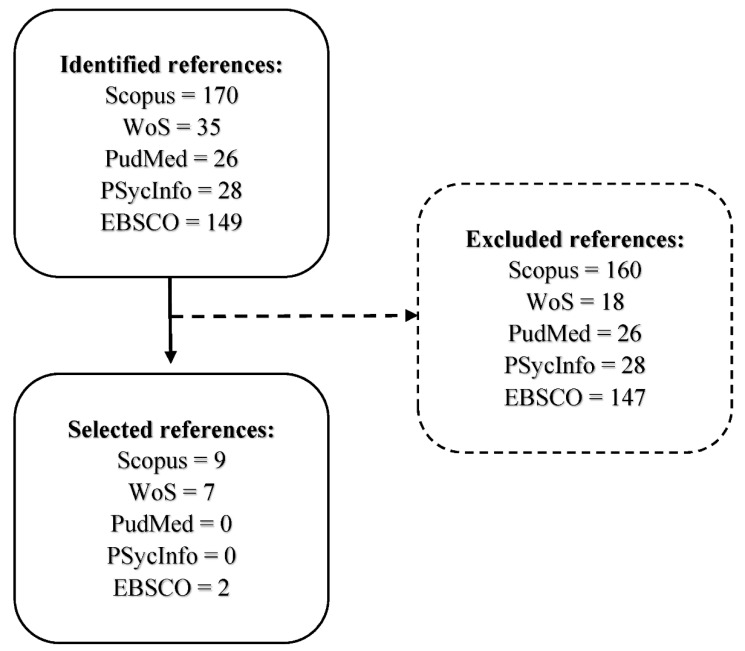
Bibliographic search flow diagram.

The search in the different databases was carried out according to the following descriptors: “intellectual disability OR intellectual functional disability OR intellectual development disorder AND body expression OR corporal expression OR dramatization OR non-verbal communication OR dance”. After screening the information, only the articles that met these criteria were included in the review, obtaining a total of 18 useful articles. Figure 1 shows the flow diagram of the literature search carried out. Those articles that were repeated and had been reviewed and counted in previous databases were not recorded for the final number of selected articles (i.e., 8 articles have been repeated up to 25 times in the different databases, thus the count of those selected was 8, with 25 exclusions).

### 2.3. Statistical Analysis

The interventions reviewed presented different results according to various categories, thus they were organized in two groups, one for those projects showing benefits related to physical condition and another group for those interventions where variables related to improvements in psychosocial factors were analyzed. 

## 3. Results

### 3.1. Quality of Interventions

To judge the methodological quality of the studies reviewed, a three-criteria quality system was applied: size of the study population, presence of a control group and/or use of systematized methodological instruments, and measurement recorded after more than one week of intervention. Margins of 10 and 30 participants were taken into account for the size of the study population. A study with a population of 30 or more participants is less vulnerable to the influence of a non-standardized sample distribution [39]. Because the number of participants in the interventions analyzed were generally quite small, an extra margin was added to make a distinction between small <10 samples and samples between 10 and 30 participants. Another criterion was to positively score the inclusion of a control group since it is important to know the effect that the intervention has had [40]. The assignment of points according to this three-factor system is shown in Table 1. The total quality of the studies may range from 1 to 5. The scores were considered low between 1 and 2, moderate between 3 and 4, and high when a 5 was obtained. Table 2 shows the quality of each of the studies reviewed.

### 3.2. Overview of the Interventions Analysed

Table 3 provides an overview of the different interventions reviewed with information on different variables of interest (i.e., number of women and men, age, study design, objectives, duration, characteristics of the intervention, variables and results) for each of the 18 articles that were analyzed. 

## 4. Discussion

The results of this study show that the performance of CE programs by people with ID is related to an improvement in their physical condition and in different psychosocial aspects for this population. There are currently several systematic reviews of sports programs for people with ID [37,59,60]. Nevertheless, there is no evidence of systematic reviews that analyze different CE programs for this group. Therefore, the main objective of this study was to review and evaluate the existing scientific literature on CE programs for people with ID. 

This review assessed 18 interventions that met the established criteria. The participants in each of these studies were people with Down syndrome, ASD and other syndromes. Related to the type of ID of the participants, the different studies explain the syndromes, of which seven studies do not indicate it, five present participants with a medium or moderate ID, one with medium to deep ID and one with severe ID, four with Down Syndrome (DS) and two studies with ASD, one of which with other syndromes too. Of these studies, nine analyzed aspects related to physical condition, motor skills and/or knowledge of one’s own body, seven considered psychosocial variables and two evaluated both aspects.

On the one hand, among the studies that focus on the improvement in physical condition in a generic way, three analyzed the improvement in balance [48,53,58]; four dealt with knowledge of one’s own body (bodily mobility, perception, postural control and/or body expression) [42,44,45,47]; two evaluated basic physical skills [49,56]; and three studied basic physical abilities, taking into account aspects such as Body Mass Index (BMI), endurance and jumping [52], strength and flexibility [46] and caloric expenditure [51].

On the other hand, articles that analyze variables related to psychosocial aspects highlight: the improvement in self-concept, emotional and personal well-being [41,43,44,55]; improving social relations and inclusion [41,54,55,57]; and mastering aspects of verbal communication [50].

The studies by Barnet et al. (2016b) and Dunphy and Hens (2018) should be highlighted because they considered both physical condition and psychosocial factors in their interventions. Various studies have shown that improvement in physical condition are often accompanied by an improvement in psychosocial aspects and vice versa [61,62]. 

Furthermore, the study by Barnet et al. (2016a) should be underlined due to the maximum score obtained in terms of the quality of the intervention carried out (i.e., 5 out of 5). This is a quantitative research where the intervention shows the psychosocial benefits of a Dance-Motion Therapy (DMT) program applied to people with ID. In this work the participants were randomly distributed: in the Intervention Group (IG) there were 22 participants (12 men/10 women) with a mean age of 47.27 ± 11.67 years, and in the Control Group (CG) there were 20 participants (12 men/8 women) with a mean age of 48.15 ± 12.46 years.

The program was organized in 26 sessions comparing the IG and the CG. The DMT program was implemented during 3 months, with two sessions per week, each lasting 1 h. The work components of the proposal dealt with body scheme, rhythm, self-concept, relationships, identification of different types of emotion, effort, balance and coordination, rooting and free dance.

The results of the application of the program pointed to changes at the emotional level emphasizing an improvement in interpersonal relationships, self-concept, reduction in anxiety, self-confidence and the ability to identify emotions, as well as the body’s self-awareness, which indicates an improvement in emotional well-being. The application of DMT leads to a greater integration of body image. Indicators on the ability to relate to other participants were also improved. Nevertheless, it should be noted that there was no change in the control group. This study concludes that the DMT program supports the emotional integration of the person, allows participants to explore new ways of communicating and expressing their own emotions and provides quality of life benefits. 

Likewise, the study by Trowslade and Hayhow [57] is relevant for three reasons: one, because it is a longitudinal qualitative study (5 years long); two, because it addresses a case study within the research itself; and three, in terms of the quality of the intervention carried out, obtaining a score of 4 out of 5. The program used mime or dramatization as a theatrical technique inspired by the renowned Open Theatre [63]. These techniques have been used to engage children emotionally, imaginatively, and corporeally through acting and expressing feelings and ideas. This intervention program aimed to improve cognitive, communication, physical, emotional and social development in children aged 3 to 11 with learning disabilities and autism.

The results reveal that the application of mime and dramatization positively influenced the children in facing risks, solving problems, being committed to their learning, being willing to reflect on their behavior and progress, and helping them think of new and imaginative ways to participate. At the same time, the study highlights the change in the teachers’ perception of how to label these students. The results also suggest that mime and dramatization provide a lively and dynamic process through which children can develop aspects related to their self-realization and diverse abilities. Finally, the case study showed that the educational community developed support and recognition towards people with autism, considering them as having multiple capacities that enrich the collective. The contributions of this research underline that the expressive work with this type of population must be oriented to their capacities and not to their deficit.

Some limitations found in this study were: (a) having used specific search tools (i.e., WoS, Scopus, PubMed, Psychoinfo and EBSCO) there is the possibility of loss of research items; (b) studies written in English and Spanish from 2010 to present were taken into account, which excludes the possibility of previous research or research in other languages; (c) some research had a small number of participants; (d) the inclusion and exclusion criteria, in order to focus the analysis on CE and ID, closed the field of review by excluding research that focused on interventions in the sports field, on medical, biomechanical aspects, in the field of virtual communication, etc. (e) Finally, studies linking physical condition, motor skills and knowledge of one’s own body together with psychosocial variables are limited in number.

## 5. Conclusions

The analysis carried out shows the potential of CE as a means of improving and developing motor, communication and relational skills in people with ID. The systematic review highlights the need for further studies to carry out CE interventions in this population. The quality of these needs improving by increasing the number of participants and the time spent on the programs. Similarly, we should consider that interventions need social and community participation to modify the perception of ID. To conclude, the importance of working with people with ID as a means of improving their inclusion in society and their quality of life should be stressed. This review will help in the design of future CE programs aimed at this population.

## Figures and Tables

**Table 1:** Evaluation of the methodological quality of the studies analysed according to three items.

	Item	Criteria	Rating
1	Number of participants	≥30 ≥10–30 ≥10	3 2 1
2	Presence of a control group/systematized methodological instruments	Yes No	1 0
3	Measures after more than one week	Yes No	1 0

**Table 2:** Quality of the studies reviewed.

Author (Year) [Reference]	Quality
Aujla & Needham-Beck (2019) [41]	3
Barnet-López et al. (2015) [42]	3
Barnet-López et al. (2016a) [43]	5
Barnet-López et al. (2016b) [44]	2
Chen, Bellama, Ryuh, & Ringenbach (2019) [45]	2
DiPasquale & Kelberman (2018) [46]	3
Dunphy & Hens (2018) [47]	4
Fotiadou, Neofotistou, Giagazoglou & Tsimaras (2017) [48]	4
Hong & Kim (2019) [49]	2
Icht (2019) [50]	4
Ito, Hiramoto & Kodama (2017) [51]	4
Martínez-Aldao, Martínez-Lemos, Bouzas-Rico & (2019) [52]	3
Massó-Ortigosa, Gutiérrez-Vilahú, Costa-Tutusaus, Oviedo, & Rey-Abella (2018) [53]	4
Montilla-Reina (2019) [54]	2
Muñoz-Moreno, Smith & Duarte (2020) [55]	3
Thergaonkar & Daniel (2019) [56]	4
Trowsdale & Hayhow (2013) [57]	4
Tsimaras, Giamouridou, Kokaridas, Sidiropoulou & Patsiaouras (2012) [58]	4

**Table 3:** Characteristics of the studies reviewed.

Authors (Year) [Reference]	Participants (*N*; Gender; Mage)	Sample Characteristics	Characteristics (Frequency; Length; Period)	Instrument	Kind of Variables	Average Improvement Score (95% IC)
Aujla & Needham-Beck (2019) [41]	*N* = 13 11 W, 2 M; Mage = 19.77		1x week; 90 min; 8–9 months	Questionnaire PWI-ID	PS	Personal and social well-being improves
Barnet-López et al. (2015) [42]	*N* = 22 12 M, 10 W Mage = 47.3		2x week; 60 min; 3 months	HFD test	PS	Emotional well-being: Improves 22.7–31.8% *p* < 0.001
Barnet-López et al. (2016a) [43]	*N* = 42, CG = 22 M, 10 W, Mage = 47.27: EG = 20 M, 8 W, Mage = 48.15	N DS = Not specified medium ID	2x week; 60 min; 3 months	HFD test	PS	Emotional well-being EG improves *p* < 0.01 CG no sig.
Barnet-López et al. (2016b) [44]	*N* = 1; 1 W; 39 years	DS	60 min; 19 sessions	Notes	PC, PS	Improves body self-concept, impulse control, initiative and communication
Chen et al. (2019) [45]	*N* = 20; SD10 Mage = 23.1 WS10 Mage = 6.7	N DS = 10 N WS = 10	3x week; 12 weeks	Camera New scale	PC	DS > SS Asymmetric movements 0.31 ** Trunk movement 0.25 * Postural control 0.17 *
DiPasquale & Kelberman (2018) [46]	*N* = 17; 10 W, 7 M; Mage = 36.82		2x week; 60 min; 12 weeks	Dynamometer (Microfet2) knee extension test (KET) Timed Up and Go test (TUG) 30-Second Sit-To-Stand test (STS) Functional reach test (FRT)	PC	Improved strength and flexibility *p* < 0.01; *p* < 0.001
Dunphy & Hens (2018) [47]	*N* = 12; DI *N* = 11 Staff *N* = 12 parents	ID ≤ 70 *N* = 12	16 weeks	Camera MARA	PC PS	Corporal Mov. 0.80 (0.59–0.91) Social Hab. 0.64 (0.30–0.84)
Fotiadou et al. (2017) [48]	*N* = 20; Mage = 10.3;	medium ID; N CG = 10 N EG = 10	2x week; 40 min; 16 weeks	Griffiths No. II questionnaire Total Balance Test	PC	Balance EG improves *p* < 0.05 CG no sig.
Hong & Kim (2019) [49]	*N* = 8		2x week; 10 weeks	Laban Movement analysis	PC	Physical abilities improve
Icht (2019) [50]	*N* = 12; Mage = 30	N CG = 6 N EG = 6	6x week; 40 min; 6 weeks	Voice recorder	PS	Verbal Communication EG improves *p* = 0.03 CG no sig.
Ito, Hiramoto & Kodama (2017) [51]	*N* = 36; 14 M, 18 W; Mage = 13.7	N DS = 8 N ASD = 9 N OS = 15	1-2x week; 20 min; 4 months; 22 sessions	Acelerómetro Camera New scale 4 criteria	PC	Calorie expenditure F(3, 31) = 24.3 ** Performance: F(3, 31) = 112.1 **
Martínez-Aldao, Martínez-Lemos, Bouzas-Rico & Ayán-Pérez (2019) [52]	*N* = 30; 17 W, 13 M; Mage = 36.37	medium DI 13, moderate 16, severe 1	2x week; 60 min; 10 weeks	Tape measure and scale ‘6-min walk test’ ‘standing long jump test’	PC	Body Mass Index −1.91 (0.15–0.93) * Endurance 4.85 (−44.86;−8.54) * Jump 24.34 (−23.50;−11.30) **
Massó-Ortigosa et al. (2018) [53]	*N* = 22; DS Mage = 20.55; WS Mage = 20.27	N DS = 11 N WS = 11	2x week; 90 min; 18 weeks	Medical history Electromyographs	PC	No significant differences
Montilla-Reina (2019) [54]	*N* = 19; DI = 3 W, 1 M; 16–21 years	N ID = 4	1-2x week; 120 min; 20 months	Participant observation	PS	Inclusion improves
Muñoz-Moreno, Smith & Duarte (2020) [55]	*N* = 42	N moderate ID = 7	9 sessions; 90 min	Participant observation	PS	Improves self-concept and socialization
Thergaonkar & Daniel (2019) [56]	*N* = 6 5–13 years	medium-moderate ID	9 months	BASIC-MR Scale ABT Scale	PC	Basic physical skills improve
Trowsdale & Hayhow (2013) [57]	*N* = 25–112 Between 3–11 years	deep to medium ID ASD	5 years, weekly	New scale Pictorial materials Portfolio	PS	Improves creative learning and social skills
Tsimaras, Giamouridou, Kokaridas, Sidiropoulou & Patsiaouras (2012) [58]	*N* = 17; CG Mage = 18.0; EG Mage = 18.1;	medium ID N CG = 7 N EG = 10	3x week; 45 min; 16 weeks	Balance Test	PC	Balance Better at EG vs. CG

*Note*. DS = Down Syndrome; WS = Without Syndrome; ASD = Autism Spectrum Disorder; OS = Other Syndromes; CG = Control Group; EG = Experimental Group; PC = Physical Condition; PS = Psycho-Social. * *p* < 0.05; ** *p* < 0.01.
